# Peer Review of “Beyond Expected Patterns in Insulin Needs of People With Type 1 Diabetes: Temporal Analysis of Automated Insulin Delivery Data”

**DOI:** 10.2196/67404

**Published:** 2024-11-27

**Authors:** Anonymous

**Keywords:** multivariate time series, k-means, clustering, machine learning, temporal patterns, data-driven, OpenAPS, open dataset, type 1 diabetes, insulin needs

*This is a peer-review report for “Beyond Expected Patterns in Insulin Needs of People With Type 1 Diabetes: Temporal Analysis of Automated Insulin Delivery Data.”*

## Round 1 Review

### General Comments

Degen et al [1] present the results of time series data derived from the OpenAPS data commons. The paper represents an important contribution to the field of diabetes technology research, as most of the work so far focused on clinical outcome analysis only. Pattern analysis of the device data provides useful insights for the entire open science community around open-source automated insulin delivery (AID) and will help researchers to identify their next research questions.

There are few papers on temporal patterns in AID research, which is why I support the publication of this comprehensive and well-written report.

### Specific Comments

1. Did the authors analyze any demographics from participants? This would be essential to exclude selection bias or at least highlight limitations if the sample is not representative of most users of open-source AID.
2. How many individual datasets were analyzed, and what is their total time span?
3. I suggest using “interstitial glucose” or “sensor glucose” versus blood glucose as continuous glucose monitoring sensors are usually placed subcutaneously and therefore do not measure (capillary) blood glucose.

## Abbreviations

AID: automated insulin delivery
